# Relationship between obstructive sleep apnea-hypopnea syndrome and osteoporosis adults: A systematic review and meta-analysis

**DOI:** 10.3389/fendo.2022.1013771

**Published:** 2022-11-17

**Authors:** Chaoyu Wang, Zhiping Zhang, Zhenzhen Zheng, Xiaojuan Chen, Yu Zhang, Chunhe Li, Huimin Chen, Huizhao Liao, Jinru Zhu, Junyan Lin, Hongwei Liang, Qiuying Yu, Riken Chen, Jinhua Liang

**Affiliations:** ^1^ Department of Pulmonary and Critical Care Medicine, The Second Affiliated Hospital of Guangdong Medical University, Zhanjiang, Guangdong, China; ^2^ Department of Pulmonary and Critical Care Medicine, Taishan Hospital of Traditional Chinese Medicine, Jiangmen, Guangdong, China; ^3^ Department of Pulmonary and Critical Care Medicine, The People's Hospital of JiangMen (Jiangmen Hospital, Southern Medical University), Jiangmen, China; ^4^ Medical College, Jiaying University, Meizhou, Guangdong, China; ^5^ State Key Laboratory of Respiratory Disease, National Clinical Research Center for Respiratory Disease, Guangzhou Institute of Respiratory Health, The First Affiliated Hospital of Guangzhou Medical University, Guangzhou, Guangdong, China; ^6^ Department of Critical Care Medicine, The First Affiliated Hospital of Guangzhou University of Chinese Medicine, Guangzhou, Guangdong, China; ^7^ Department of Traditional Chinese Medicine, The Second Affiliated Hospital of Guangdong Medical University, Zhanjiang, Guangdong, China; ^8^ Department of Endocrinology, The Second Affiliated Hospital of Guangdong Medical University, Zhanjiang, Guangdong, China

**Keywords:** obstructive sleep apnea-hypopnea syndrome, meta-analysis, osteoporosis, bone density, lumbar spine

## Abstract

**Objective:**

This study is undertaken to explore the relationship between obstructive sleep apnea-hypopnea syndrome (OSAHS) and osteoporosis, including the relationship between OSAHS and osteoporosis incidence, lumbar spine bone mineral density (BMD), and lumbar spine T-score.

**Method:**

Cochrane Library, PubMed, Embase, Web of Science, and other databases are searched from their establishment to April 2022. Literature published in 4 databases on the correlation between OSAHS and osteoporosis,lumbar spine BMD,lumbar spine T-score is collected. Review Manager 5.4 software is used for meta-analysis.

**Results:**

A total of 15 articles are selected, including 113082 subjects. Compared with the control group, the OSAHS group has a higher incidence of osteoporosis (OR = 2.03, 95% CI: 1.26~3.27, Z = 2.90, P = 0.004), the lumbar spine BMD is significantly lower (MD = -0.05, 95% CI: -0.08~-0.02, Z = 3.07, P = 0.002), and the lumbar spine T-score is significantly decreased (MD = -0.47, 95% CI: -0.79~-0.14, Z = 2.83, P = 0. 005).

**Conclusion:**

Compared with the control group, the OSAHS group has a higher incidence of osteoporosis and decreased lumbar spine BMD and T-score. In order to reduce the risk of osteoporosis, attention should be paid to the treatment and management of adult OSAHS, and active sleep intervention should be carried out.

## 1 Introduction

Obstructive sleep apnea-hypopnea syndrome (OSAHS) is a sleep disorder characterized by recurrent episodes of apnea that lead to hypoxia, hypercapnia, and sleep disruption (1). Osteoporosis is a bone metabolism disorder characterized by decreased bone mass, destruction of bone microstructure, and susceptibility to fractures (2). It is generally believed that OSAHS is associated with a higher incidence of osteoporosis (3–5), and spinal deformity is one of its main clinical manifestations, as well as kyphosis, limited spinal extension, etc., causing great distress to the affected population and warranting further research. Several studies have explored the relationship between OSAHS and lumbar osteoporosis. Studies by Liguori, Chen et al. (4, 6) suggest that OSA may be a risk factor in bone mineral density (BMD), leading to osteopenia and osteoporosis. The reason may be that hypoxia slows down the growth of osteoblasts, it promotes the activation of osteoclasts. Sforza et al. (7) showed that the protective effects of intermittent hypoxia on bone metabolism, after taking into account the age-related decrease in BMD, reduced the risk of osteopenia and osteoporosis in elderly people with OSAHS. The results of these studies are inconsistent, which has not only caused great trouble for clinicians, but also affected the prevention and treatment of lumbar osteoporosis in patients with OSAHS. The purpose of this study is to conduct a meta-analysis of existing clinical studies so as to explore the relationship between OSAHS and the occurrence of lumbar osteoporosis and BMD changes, thereby providing evidence-based prevention and intervention for lumbar osteoporosis in patients with medical evidence of OSAHS.

## 2 Materials and methods

### 2.1 Retrieval strategy

According to the Meta-analysis of Observational Studies in Epidemiology (MOOSE) statement (8) and the Systematic Review and Meta-analysis report (Preferred Reporting Items for Systematic) Reviews and Meta-Analyses, PRISMA) standard (9). Cochrane Library, PubMed, Embase, and Web of Science databases were searched from their establishment to April 2022. English search terms included Sleep apnea, obstructive, Obstructive Sleep Apnea, Sleep Apnea Hypopnea syndrome, Sleep-related breathing disorder, Osteoporosis, Bone density, Bone mass, Bone loss and Osteo. The protocol was registered in the Prospective Register of Systematic Reviews(Prospero CRD42022339017).

### 2.2 Literature inclusion and exclusion criteria

Inclusion criteria (1): the subjects of the study were adults over 18 years of age (2); the article types were cohort studies, case-control studies, and cross-sectional studies, observing BMD and T-score in patients with sleep apnea or obstructive sleep apnea, evaluating the incidence or prevalence of osteoporosis, and comparing them with the control group (3); OSAHS was diagnosed by polysomnography or portable sleep monitor, and the severity was evaluated by the apnea hypopnea index (AHI), which is the sum of the average number of apnea and hypopnea events per hour (10) (4); lumbar spine BMD (measured in g/cm^2^) and/or T-score were measured by dual energy X-ray densitometer, and osteoporosis was defined as BMD and T-score < -2.5 SD (11) (5); based on different reports from the same research population, the articles with the largest sample sizes were included.

Exclusion criteria (1): languages other than English (2); studies without a control group (3); studies where the effect size cannot be extracted or calculated (4); studies for which the authors did not respond to contact or could not provide meta-analysis data (5); application of glucocorticoids or other drugs that affect BMD.

### 2.3 Literature screening, quality assessment, and data extraction

Two researchers independently searched, extracted and screened the literature, checked each other’s work, and provided literature with differences to the third researcher for analysis to decide whether or not it should be included. The methodological quality of the included literature was assessed. The quality of the included studies was assessed using the Newcastle-Ottawa Scale (NOS) (12). Only high-quality articles rated higher than 6 stars were included.The extracted data included the first author, study area, publication time, study type, sample size, age, AHI, OSAHS assessment method, BMD, T-score, outcome measures, and adjustment for confounders. After data extraction, the data was checked, and inconsistent data was extracted again. After checking, the data was analyzed.

### 2.4 Ending and exposure

The lumbar spine BMD (measured in g/cm^2^) and/or T-score of the subjects were obtained by dual-energy X-ray densitometry, and OSAHS was diagnosed by polysomnography or portable sleep monitor. The incidence of osteoporosis, BMD, and lumbar spine T-score in the OSAHS group and control group were used as outcome indicators. The difference in the incidence of osteoporosis between the OSAHS group and control group indicated the correlation between OSAHS and osteoporosis; the difference in BMD between the OSAHS group and control group indicated the effect of OSAHS on BMD; when the OSAHS group was compared with the control group, the level of lumbar spine T-score was different, indicating the influence of OSAHS.

### 2.5 Statistical methods

Statistical analysis was performed using Review Manager 5.4 software. MD and OR values were used for effect evaluation, and 95% CI was calculated. The heterogeneity of the studies was analyzed using the I^2^ statistic test and Q test. I^2^ < 50% and P > 0.1 indicated no significant heterogeneity among the studies, while I^2^ > 50% and P < 0.1 indicated statistical heterogeneity. If there is obvious heterogeneity, the random effect model is used for analysis. Sensitivity analysis can also be conducted to eliminate articles with obvious heterogeneity, and then fixed effect model meta-analysis can be conducted. The presence of publication bias was estimated by funnel plot and Egger’s test. For the analysis results with heterogeneity, the included studies will be stratified according to differences in countries and regions, population age differences, gender, and OSAHS severity for subgroup analysis.

## 3 Results

### 3.1 Literature screening results

**Figure 1:**
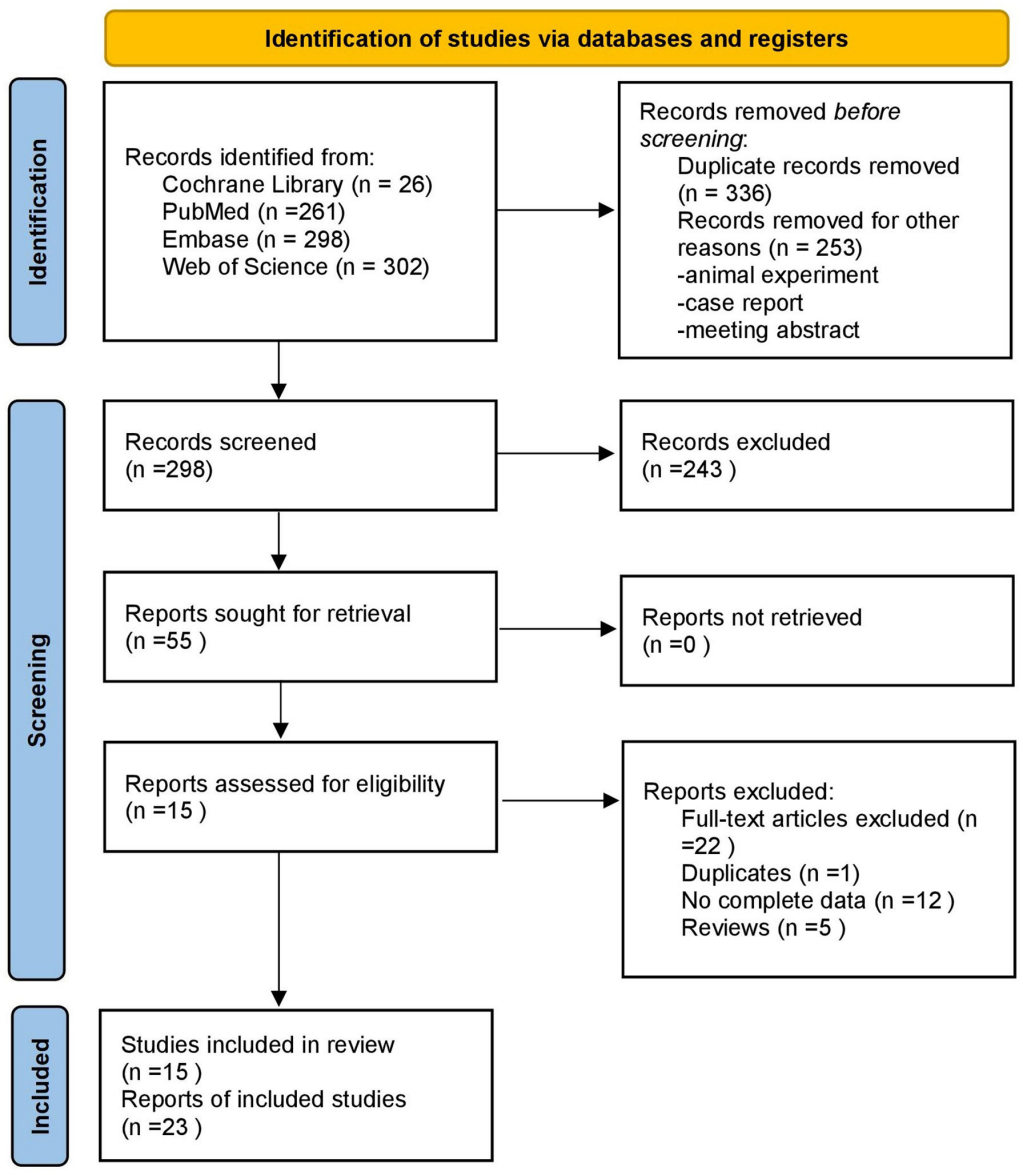
Flow diagram of literature screening.

A total of 887 articles were retrieved, 603 were obtained after deduplication, 248 were excluded by reading the titles and abstracts, and 15 were finally included after reading the full text (**Figure 1**). The study populations were from China; Taiwan, China; Turkey; Croatia; Italy; and France. The basic characteristics of the literature included in the study are shown in **Table 1**.

**Table 1:** Basic features of the included studies.

Study	Countryregion	Age	Sample size(/n)	Criteria for OSA	Gender	Research object characteristics
Uzkeser2013 (3)	Turkey	54(37~69)	47	AHI>10events/h	Male	no concomitant disease
Sforza2013 (7)	France	68.6±0.8	832	AHI≥15events/h	M/F	Concomitant DM and HTN
Yen2014 (13)	Taiwan,China	48.9±14.5	90226	ICD-9-CM	M/F	Concomitant DM,HTN,COPD, cancer,etc
Chen2014 (4)	Taiwan,China	>40	21032	ICD-9-CM	M/F	Concomitant DM,HTN, CHD,cancer,etc
ASLAN2015 (14)	Turkey	48.5(40~68)	46	AHI≥6events/h	Male	no concomitant disease
Yuceege2015 (15)	Turkey	35.5±50.7	85	AHI≥15events/h	M/F	no concomitant disease
Terzi2015 (16)	Turkey	52.37±8.58	50	AHI≥5events/h	Male	Concomitant HTN
Wang2015 (17)	Taiwan,China	71.6±8.5	66	AHI≥15events/h	M/F	Concomitant COPD
Liguori2016 (6)	Italy	51.72±11.82	142	AHI>15events/h	Male	no concomitant disease
Chen2017 (18)	China	42.44±11.84	84	AHI≥10events/h	Male	no concomitant disease
Qiao2018 (19)	China	30~65	119	AHI≥5events/h	Male	no concomitant disease
Pazarli2018 (20)	Turkey	48.55±11.8	89	AHI≥5events/h	M/F	no concomitant disease
Ma2018 (21)	China	18~60	68	AHI≥5events/h	Male	no concomitant disease
Vilovic2020 (22)	Croatia	20~65	103	AHI≥15events/h	Male	no concomitant disease
Sadaf2021 (23)	Turkey	48.02±8.435	93	AHI>5events/h	M/F	no concomitant disease

OSA, Obstructive sleep apnea; M/F,Male to Female; BMI, Body Mass Index; CHD,coronary heart disease; DM, Diabetes mellitus; HTN, High Twisted Nematic; COPD, Chronic obstructive pulmonary diseases;

ICD-9-CM, CM International Classification of Diseases, Ninth Revision, Clinical Modification.

### 3.2 Quality assessment of included studies

The quality of the included observational studies was assessed using the NOS scale, which is shown in **Table 2**. The lowest overall rating was 6★ and the highest was 7★, all moderate to high quality, with low to moderate risk of bias, and no studies were excluded for poor quality (< 5★).

**Table 2:** Newcastle–Ottawa Scale of the included studies.

Study	Year	Selection	Comparablity	Exposure	Quality scores
Uzkeser	2013	★★★	★★	★★	7
Sforza	2013	★★★	★★	★	6
Yen	2014	★★★	★	★★	6
Chen	2014	★★★	★	★★	6
ASLAN	2015	★★★	★★	★★	7
Yuceege	2015	★★★	★★	★	6
Terzi	2015	★★★	★★	★	6
Wang	2015	★★★	★★	★★	7
Liguori	2016	★★★	★★	★	6
Chen	2017	★★★	★★	★	6
Qiao	2018	★★★	★★	★	6
Pazarli	2018	★★★	★	★★	6
Ma	2018	★★★	★★	★★	7
Vilovic	2020	★★★★	★★	★	7
Sadaf	2021	★★★	★★	★	6

Each ★ represents a quality score of 1 point, and the sum of all ★ is the final quality score.

### 3.3 Results

Our results include: ① the relationship between OSAHS and the incidence rate of osteoporosis; ② The relationship between OSAHS and lumbar bone mineral density; ③ The relationship between OSAHS and lumbar T-score. We describe the corresponding statistical results in detail below and we have summarized the effect size value for the mean difference of each study, as shown in **Table 3**.

**Table 3:** The effect size value for mean differences of the included studies.

Study	Year	Outcome	Results
Uzkeser	2013	Lumbar Spine BMD	Lumbar Spine BMD:MD=-0.02 (95%CI:-0.09~0.05)
		Lumbar Spine T-score	Lumbar Spine T-score:MD=-0.53 (95%CI:-1.07~-0.01)
Sforza	2013	Lumbar Spine BMD	Lumbar Spine BMD:MD=0.04 (95%CI:-0.14~0.22)
		Lumbar Spine T-score	Lumbar Spine T-score:MD=0.26 (95%CI:0.07~0.4)
Yen	2014	Osteoporosis Incidence	Osteoporosis Incidence:OR=1.60 (95%CI:1.27~2.02)
Chen	2014	Osteoporosis Incidence	Osteoporosis Incidence:OR=2.52 (95%CI:1.58~4.02)
ASLAN	2015	Osteoporosis Incidence	Osteoporosis Incidence:OR=12.69 (95%CI:0.66~244.42)
Yuceege	2015	Lumbar Spine BMD	Lumbar Spine BMD:MD=-0.08 (95%CI:-0.14~-0.02)
		Lumbar Spine T-score	Lumbar Spine T-score:MD=-0.74 (95%CI:-1.26~-0.22)
Terzi	2015	Lumbar Spine BMD	Lumbar Spine BMD:MD=-0.08 (95%CI:-0.16~-0.00)
Wang	2015	Lumbar Spine T-score	Lumbar Spine T-score:MD=-0.72 (95%CI:-1.22~-0.22)
Liguori	2016	Lumbar Spine BMD	Lumbar Spine BMD : MD=-0.13 (95%CI:-0.18~-0.08)
		Lumbar Spine T-score	Lumbar Spine T-score:MD=-1.11 (95%CI:-1.52~-0.70)
Chen	2017	Lumbar Spine BMD	Lumbar Spine BMD : MD=0.01 (95%CI:-0.09~-0.11)
		Lumbar Spine T-score	Lumbar Spine T-score:MD=005 (95%CI:-0.64~0.74)
Qiao	2018	Lumbar Spine BMD	Lumbar Spine BMD : MD=-0.01 (95%CI:-0.09~0.11)
Pazarli	2018	Lumbar Spine BMD	Lumbar Spine BMD : MD=-0.02 (95%CI:-0.04~-0.00)
		Lumbar Spine T-score	Lumbar Spine T-score:MD=-0.15 (95%CI:-0.34~0.04)
Ma	2018	Lumbar Spine BMD	Lumbar Spine BMD : MD=-0.08 (95%CI:-0.13~-0.03)
		Lumbar Spine T-score	Lumbar Spine T-score:MD=-0.59 (95%CI:-1.03~-0.15)
Vilovic	2020	Lumbar Spine BMD	Lumbar Spine BMD : MD=-0.02 (95%CI:-0.11~-0.07)
		Lumbar Spine T-score	Lumbar Spine T-score:MD=-0.23 (95%CI:-0.78~0.32)
Sadaf	2021	Lumbar Spine T-score	Lumbar Spine T-score:MD=-0.99 (95%CI:-1.43~-0.55)

BMD, Bone mineral density.

#### 3.3.1 Association of OSAHS with osteoporosis incidence

**Figure 2:**
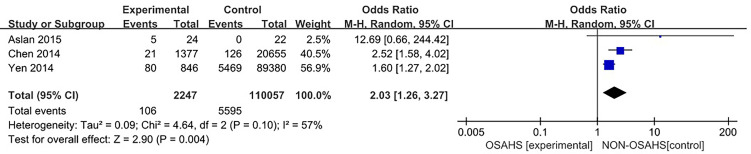
Forest plot of the incidence of osteoporosis in OSAHS group and control group.

Three studies (4, 13, 14) provided specific numbers of patients with osteoporosis among their study subjects. All three studies were included in the analysis (**Figure 2**). The results of the heterogeneity test indicated that there was statistical heterogeneity among the studies (P = 0.1, I^2^ = 57%), so a random effect model was used. The results showed that compared with the control group, the OSAHS group had a higher incidence of osteoporosis (OR = 2.03, 95% CI: 1.26~3.27, Z = 2.90, P = 0.004).

**Figure 3:**
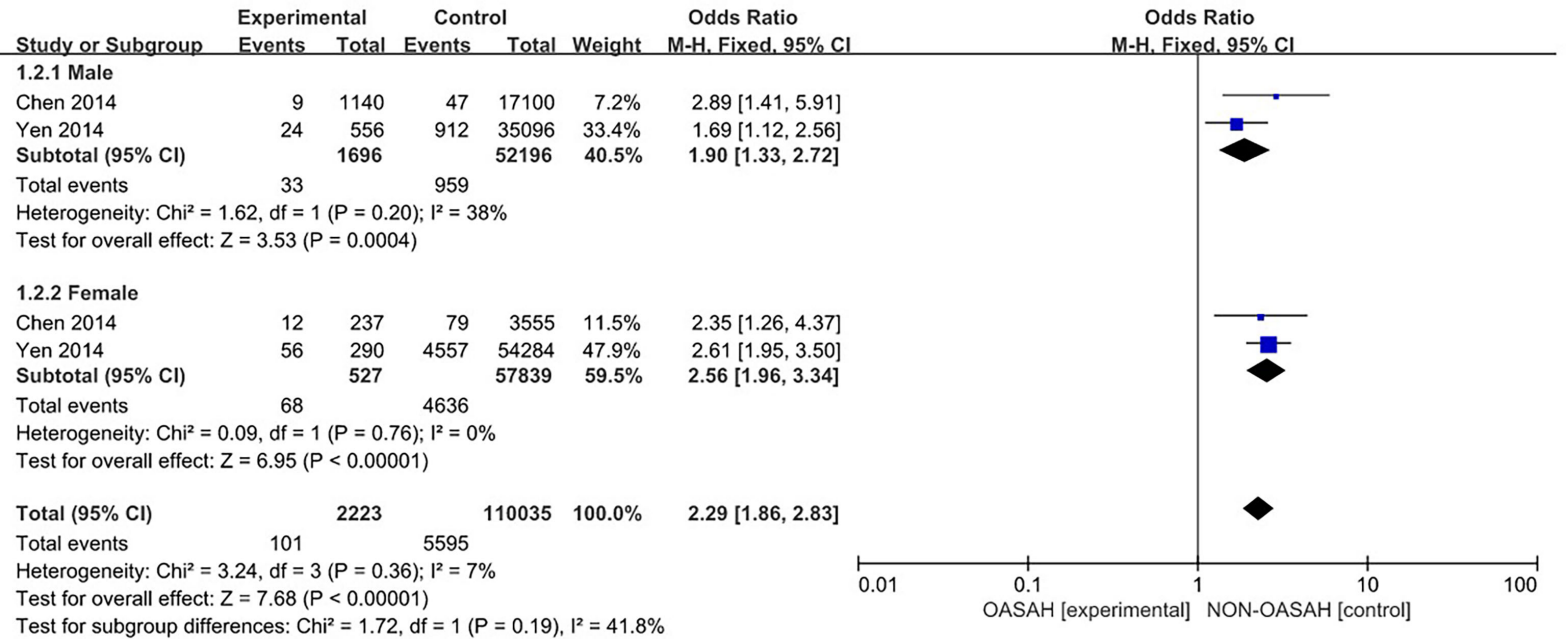
Forest plot of incidence of osteoporosis in male and female subgroups.

**Figure 4:**
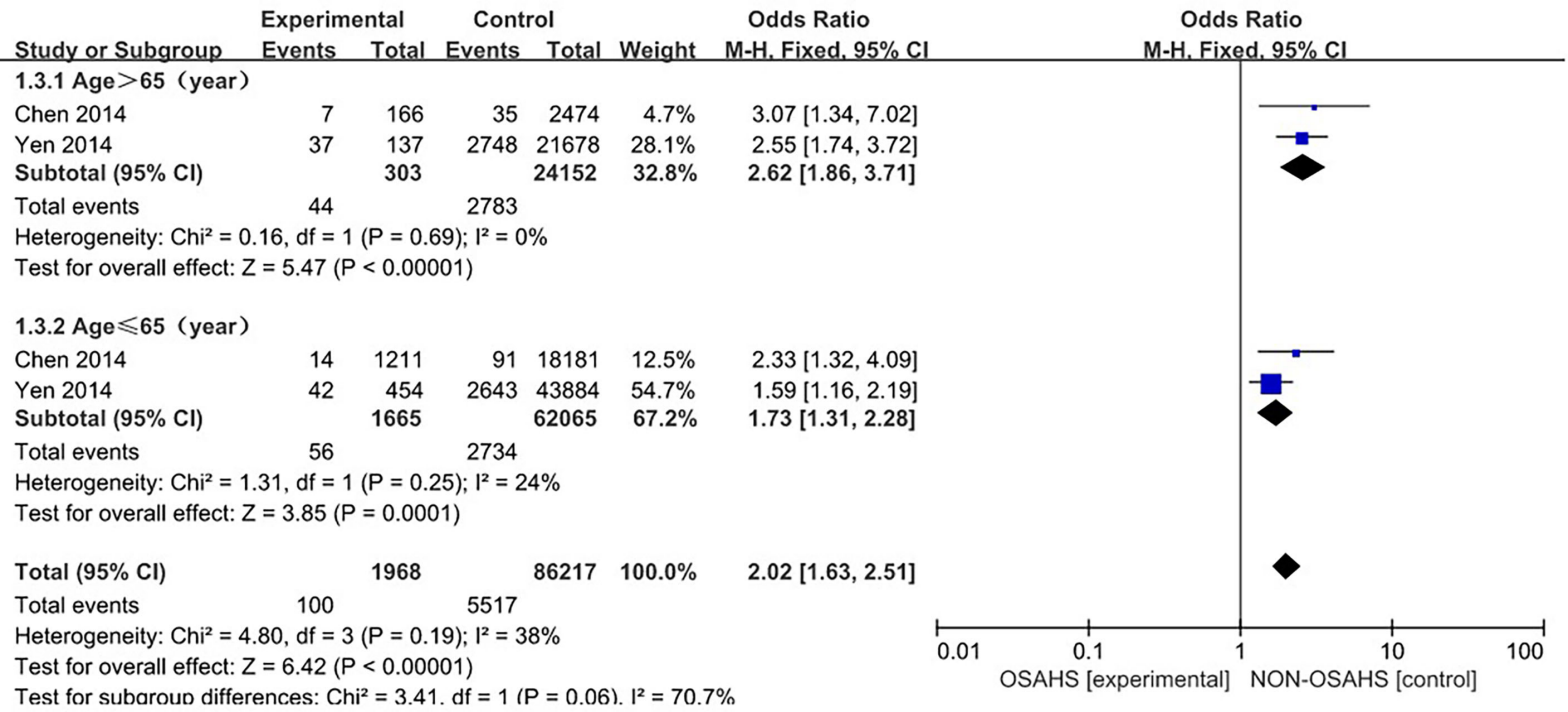
Forest plot of the incidence of osteoporosis in elderly (>65 years old) and middle-aged (40-65 years old) subgroups.

Two studies (4, 13) provided specific numbers of osteoporosis in men, women, elderly people (> 65 years), and middle-aged people (40~65 years). To reduce the clinical heterogeneity of the study subjects, subgroup analyses were performed by gender (**Figure 3**) and age (**Figure 4**). The results showed that, compared with the control group, in the gender subgroup analysis of the OSAHS group, the combined heterogeneity of the two groups was (P = 0.36, I^2^ = 7%), and there was no statistically significant heterogeneity among the male group (P = 0.36, I^2^ = 7%) = 0.2, I^2^ = 38%), female group (P = 0.76, I^2^ = 0%), so a fixed effect model was used. After gender subgroup analysis, the heterogeneity of the osteoporosis studies was significantly reduced, male (OR = 1.90, 95% CI: 1.33-2.72, Z = 3.53, P < 0.001), female (OR = 2.56, 95% CI: 1.96-3.34, Z = 6.95, P < 0.001), The incidence of osteoporosis in OSAHS group was higher and statistically significant; the combined final effect size of the gender subgroup analysis was (OR = 2.29, 95% CI: 1.86-2.83, Z = 7.68, P < 0.001); The incidence of osteoporosis in OSAHS group is high and statistically significant. In the subgroup analysis of age, the combined heterogeneity of the two groups was (P = 0.19, I^2^ = 38%), and there was slight heterogeneity in the statistics. The elderly (> 65 years old) group was (P = 0.69, I^2^ = 0%) and the middle-aged (40~65 years old) group was (P = 0.25, I^2^ = 24%), so a fixed effect model was used. After age subgroup analysis, the heterogeneity of the osteoporosis studies was significantly reduced. The elderly (> 65 years old) group was (OR = 2.62, 95% CI: 1.86~3.71, Z = 0.89, P < 0.001) and the middle-aged (40~65 years old) group was (OR = 1.73, 95% CI: 1.31~2.28, Z = 3.31, P < 0.001), so the OSAHS group had a higher incidence of osteoporosis, which was statistically significant. The combined final effect size of the age subgroup analysis was (OR = 2.02, 95% CI: 1.63~2.51, Z = 6.42, P < 0.001); the OSAHS group had a higher incidence of osteoporosis, which was statistically significant.

The forest plot analysis of OSAHS and the incidence of osteoporosis suggest that OSAHS is associated with the prevalence of osteoporosis and is a risk factor for the disease.

#### 3.3.2 Association of OSAHS with lumbar spine BMD

**Figure 5:**
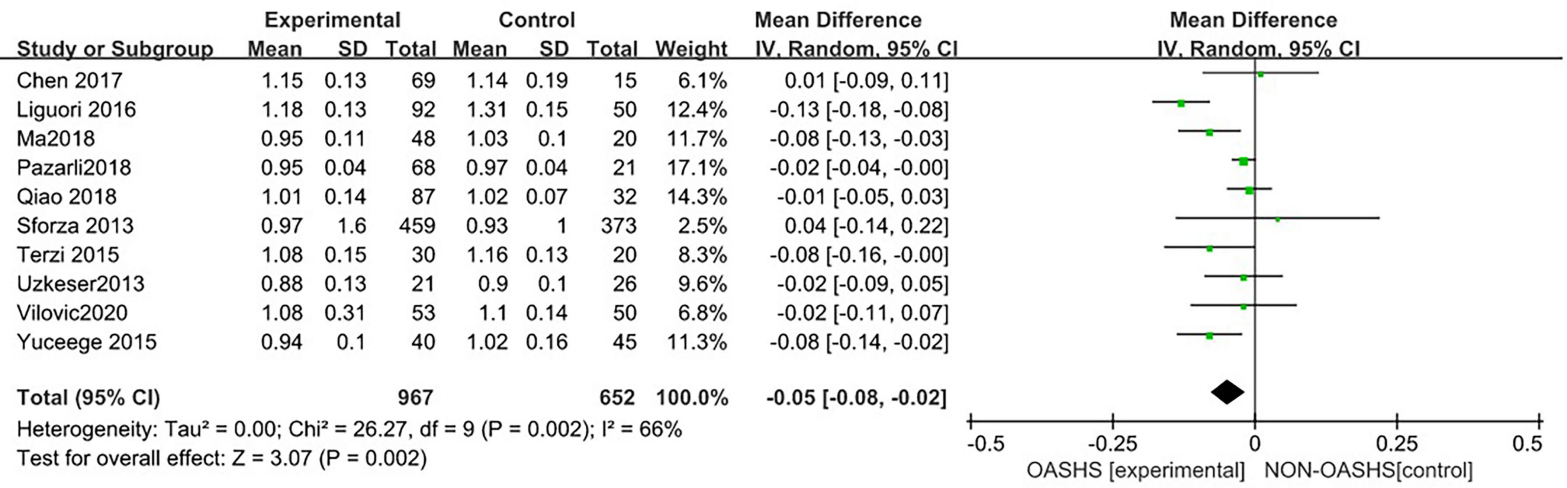
Forest plot of lumbar spine bone mineral density between OSAHS group and control group.

Ten studies (3, 6, 7, 15, 16, 18–22) were included in a meta-analysis of lumbar spine BMD (**Figure 5**). Compared with the control group, lumbar spine BMD was significantly lower in the OSAHS group (MD = -0.05, 95% CI: -0.08~-0.02, Z = 3.07, P = 0.002). There was moderate heterogeneity between studies (I^2^ = 66%, P = 0.002), so a random effect model was used. The elderly population is at increased risk for OSAHS (24, 25) due to changes in the anatomy and function of the upper airway (26), and the frequent coexistence of other medical conditions such as diabetes, hypertension, and cardiovascular disease. Meanwhile, BMD gradually decreases with age (27). Risk factors such as old age, diabetes, hypertension, and some diseases that affect osteoporosis may affect the results of osteoporosis research, leading to unstable results for the association between OSAHS and lumbar spine BMD. To further verify the relationship between OSAHS and lumbar spine BMD, and further reduce the clinical heterogeneity of the study subjects, we conducted a subgroup analysis after excluding osteoporosis-related risk factors, including a subgroup analysis of AHI grouping by OSAHS diagnostic criteria and regional subgroup analysis. The research population of Sforza2013 (7) was older than 65 and accompanied by hypertension, diabetes, and other diseases; in Terzi2015 (16), some of the research subjects had hypertension complications; and all of the above may affect the results of a lumbar spine BMD study.

##### 3.3.2.1 AHI subgroup analysis

**Figure 6:**
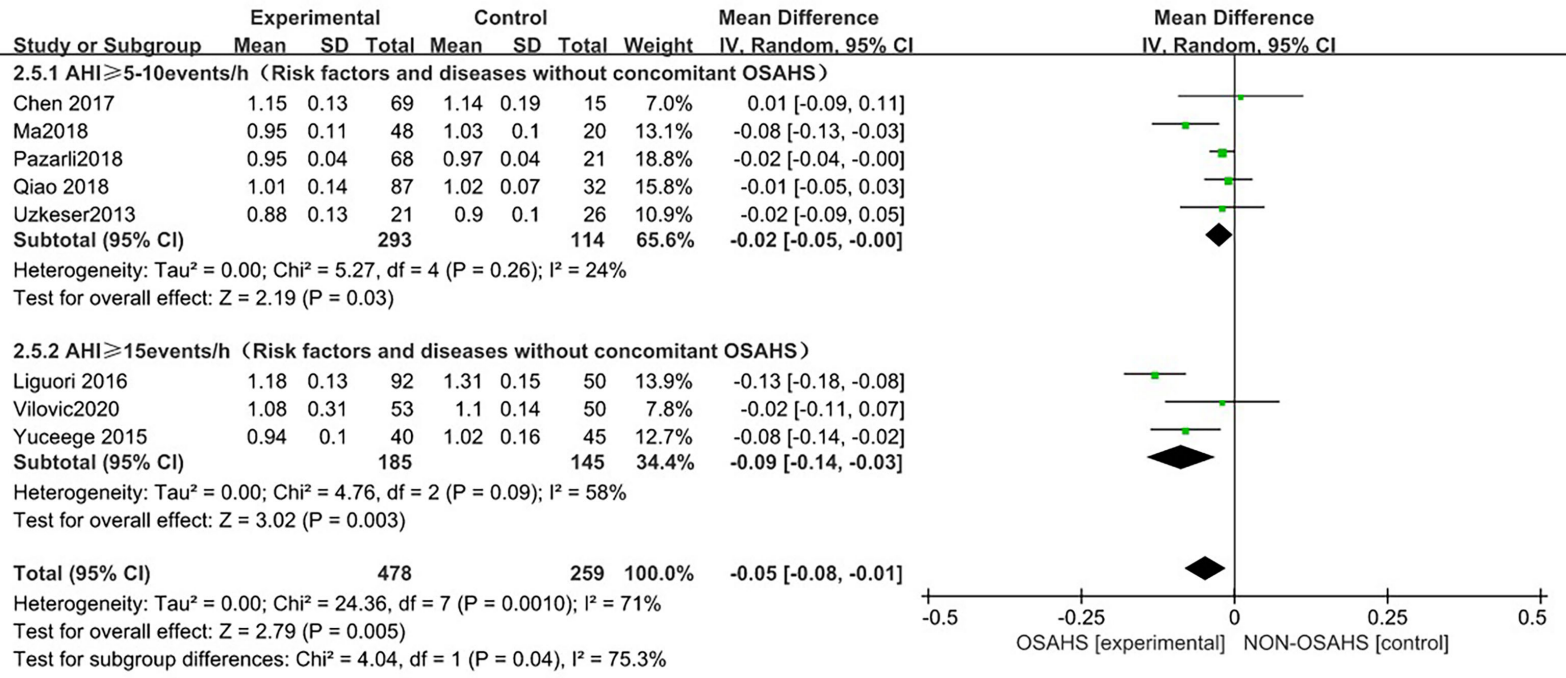
Forest plot of subgroup analysis of lumbar spine BMD in OSAHS group and control group by “AHI group” (grouped according to OSAHS diagnostic criteria, after exclusion of osteoporosis-related risk factors).

In a subgroup analysis of AHI grouping with OSAHS diagnostic criteria (after the exclusion of osteoporosis-related risk factors) (**Figure 6**), 8 studies were included (3, 6, 15, 18–22), and the groups were combined for heterogeneity (P = 0.001, I^2^ = 71%). There was moderate heterogeneity in the statistics. In the subgroup analysis, the OSAHS diagnostic heterogeneity criteria of AHI > 5~10 events/h group was (P = 0.26, I^2^ = 24%), and the grouping heterogeneity of AHI > 15 events/h was (P = 0.09, I^2^ = 58%), so a random effect model was used. After the subgroup analysis of OSAHS diagnostic criteria AHI grouping, the correlation between OSAHS and lumbar spine BMD was different. The quality was significantly reduced. The results of subgroup analysis showed that compared with the control group, the lumbar spine BMD of the OSAHS group with AHI > 5~10 events/h was slightly lower (MD = -0.02, 95% CI: -0.05~-0.00, Z = 2.19, P = 0.03), the lumbar spine BMD in the AHI > 15 events/h group was significantly decreased (MD = -0.09, 95% CI: -0.14~-0.03, Z = 3.02, P = 0.003), and the difference was statistically significant. The effect size of lumbar spine BMD in the OSAHS group AHI > 15 events/h was higher than that of the OSAHS diagnostic criteria AHI > 5~10 events/h group, indicating that in patients grouped by OSAHS diagnostic criteria AHI > 15 events/h, compared with AHI > 5~10 events/h, the risk of lumbar BMD decline was higher, so the severity of OSAHS may be related to lumbar BMD. The combined effect size of the AHI group was (MD = -0.05, 95% CI: -0.08~-0.01, Z = 2.79, P = 0.005). The OSAHS group had lower lumbar spine BMD, the results remained unchanged after excluding risk factors for osteoporosis, and the difference was statistically significant.

##### 3.3.2.2 Regional subgroup analysis

**Figure 7:**
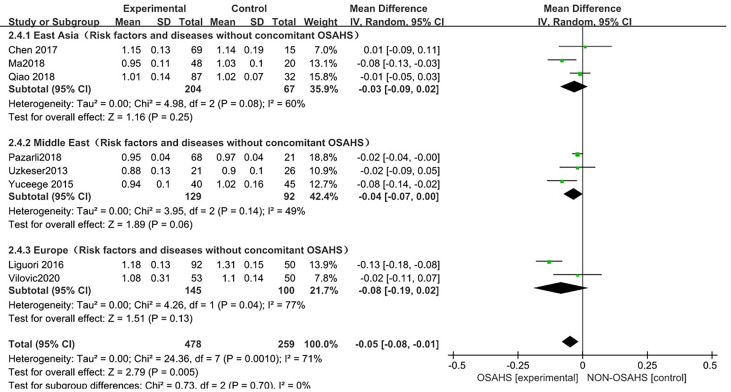
Forest plot of subgroup analysis of lumbar spine BMD in OSAHS group and control group (after excluding risk factors related to osteoporosis).

In the regional subgroup analysis (after excluding osteoporosis-related risk factors), (**Figure 7**), 8 studies were included (3, 6, 15, 18–22), and the combined group heterogeneity was (P = 0.001, I^2^ = 71%), indicating moderate statistical heterogeneity. In the subgroup analysis, the grouping heterogeneity in East Asia was (P = 0.08, I^2^ = 60%), that in the Middle East was (P = 0.14), I^2^ = 49%), and that in Europe was (P = 0.04, I^2^ = 77%), so a random effect model was used. The results of the subgroup analysis showed that, compared with the control group, the East Asian group was (MD = -0.03, 95% CI: -0.09~0.02, Z = 1.16, P = 0.25), Middle East group was (MD = -0.04, 95%). CI: -0.07~0.00, Z = 1.89, P = 0.06), and European group was (MD = -0.08, 95% CI: -0.19~0.02, Z = 1.51, P = 0.13), so the lumbar spine BMD was lower in each regional grouping, but the difference was not statistically significant. The combined effect size of the regional grouping was (MD = -0.05, 95% CI: -0.08~-0.01, Z = 2.79, P = 0.005), indicating that the OSAHS group had lower lumbar spine BMD, and the subgroup analysis was performed after excluding osteoporosis risk factors when the results remained stable and the difference was statistically significant.

The forest plot analysis of OSAHS and lumbar spine BMD studies suggested that OSAHS was associated with lumbar spine BMD, OSAHS was a risk factor for the decrease in lumbar spine BMD, and the severity of OSAHS may be related to lumbar spine BMD.

#### 3.3.3 Association of OSAHS with lumbar spine T-score

**Figure 8:**
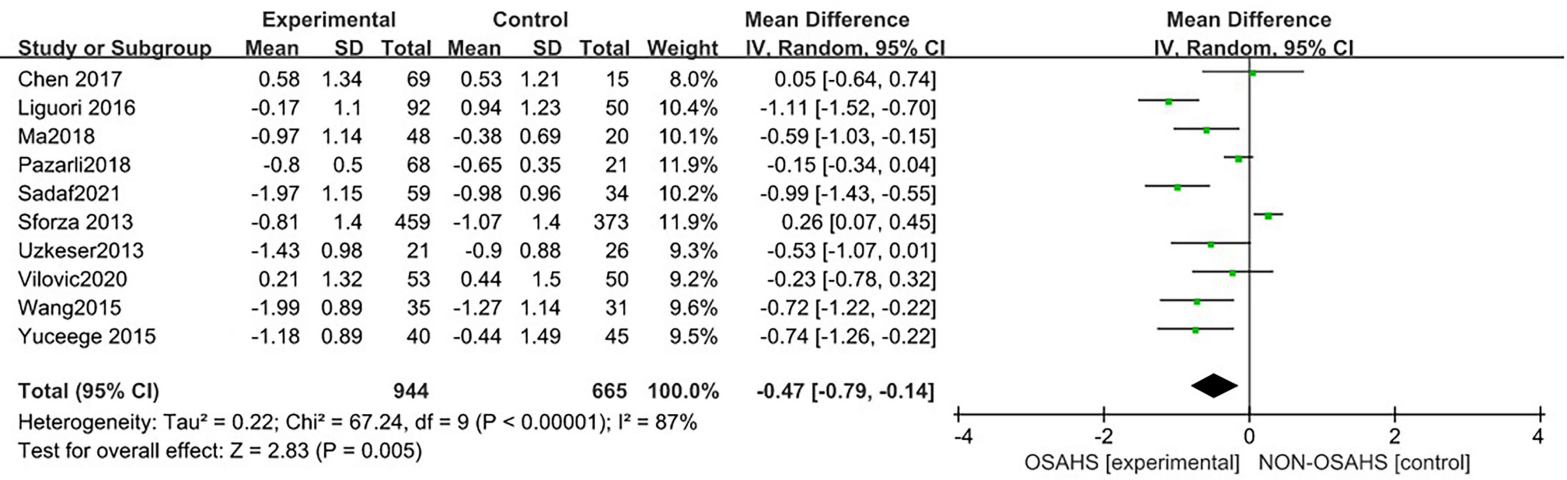
Forest plot of lumbar spine T-score between OSAHS group and control group.

Ten studies (3, 6, 7, 15, 17, 18, 20–23) were included in the meta-analysis of lumbar spine T-score (**Figure 8**). Compared with the control group, the lumbar spine T-score was significantly lower in the OSAHS group (MD = -0.47, 95% CI: -0.79~-0.14, Z = 2.83, P = 0.005). There was high heterogeneity between studies (I^2^ = 87%, P < 0.001), so a random effect model was used. Similarly, risk factors such as old age, diabetes, hypertension, and some diseases that affect osteoporosis may affect the results of osteoporosis research, leading unstable results for the association between OSAHS and lumbar spine T-score. In order to further verify the relationship between OSAHS and lumbar spine T-score, and further reduce the clinical heterogeneity of the studies, we conducted a subgroup analysis after excluding risk factors related to osteoporosis, including subgroups grouped by AHI according to the OSAHS diagnostic criteria. The research population of Sforza2013 (7) was older than 65 and accompanied by hypertension, diabetes, and other diseases; the mean age of the research population of Wang2015 (17) was more than 65 and accompanied by chronic obstructive pulmonary disease; as both may affect the results of lumbar spine BMD studies, they were not included in the subgroup analysis.

##### 3.3.3.1 AHI subgroup analysis

**Figure 9:**
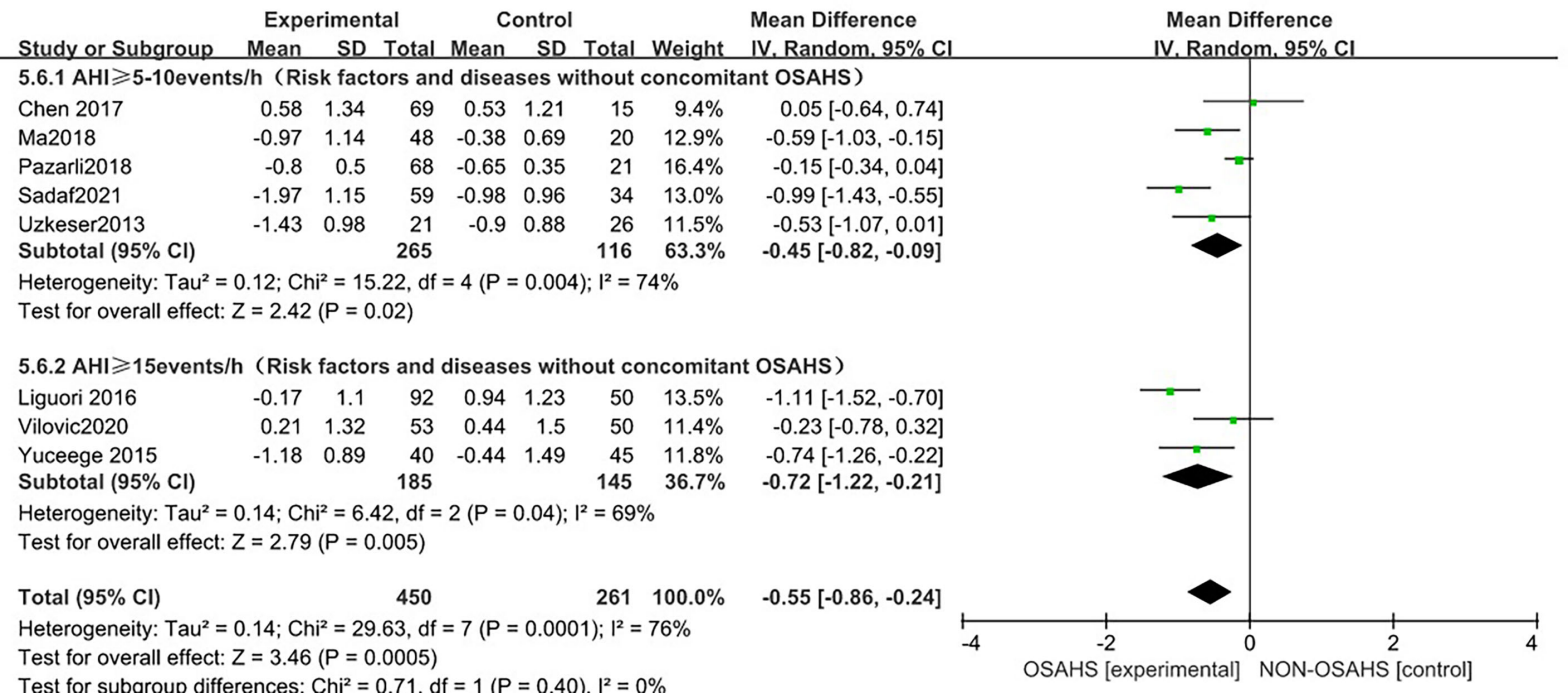
Forest plot of lumbar spine T-score “AHI grouping” subgroup analysis between OSAHS group and control group (grouped according to OSAHS diagnostic criteria, after exclusion of osteoporosis-related risk factors).

In a subgroup analysis of AHI grouping according to OSAHS diagnostic criteria (after the exclusion of osteoporosis-related risk factors) (**Figure 9**), 8 studies were included (3, 6, 15, 18, 20–23) with heterogeneous groupings. In the subgroup analysis, the grouping heterogeneity of OSAHS diagnostic criteria AHI > 5~10 events/h was (P = 0.004, I^2^ = 74%). %), and the grouping heterogeneity of AHI ≥ 15 events/h was (P = 0.04, I^2^ = 69%), so a random effect model was used. After the OSAHS diagnostic criteria AHI grouping subgroup analysis, The heterogeneity in the correlation study between OSAHS and lumbar spine T-score was significantly reduced, and the heterogeneity of AHI ≥ 5~10 events/h group and AHI ≥ 15 events/h group were reduced to moderate heterogeneity. The results of the subgroup analysis showed that compared with the control group, the lumbar spine T-score in the OSAHS AHI ≥ 5~10 events/h group was decreased (MD = -0.45, 95% CI: -0.82~-0.09, Z = 2.42, P < 0.001), the lumbar spine T-score in the AHI > 15 events/h group was significantly decreased (MD = -0.72, 95% CI: -1.22~-0.21, Z = 2.79, P = 0.005), and the difference was statistically significant. The lumbar spine T-score effect size of the OSAHS diagnostic criteria AHI ≥ 15 events/h group was higher than that of the OSAHS diagnostic criteria AHI ≥ 5~10 events/h group, indicating that compared with AHI ≥ 5-10 events/h group, The risk of lumbar T-score decline was higher in the the patients in the OSAHS diagnostic criteria AHI ≥ 15 events/h group, and the severity of OSAHS may be related to lumbar T-score. The combined effect size of the AHI group (MD = -0.55, 95% CI: -0.86~-0.24, Z = 3.46, P < 0.001), the lumbar spine T-score of the OSAHS group was also lower, excluding the risk factors related to osteoporosis. After group analysis, the results remained stable and the difference was statistically significant.

##### 3.3.3.2 Regional subgroup analysis

**Figure 10:**
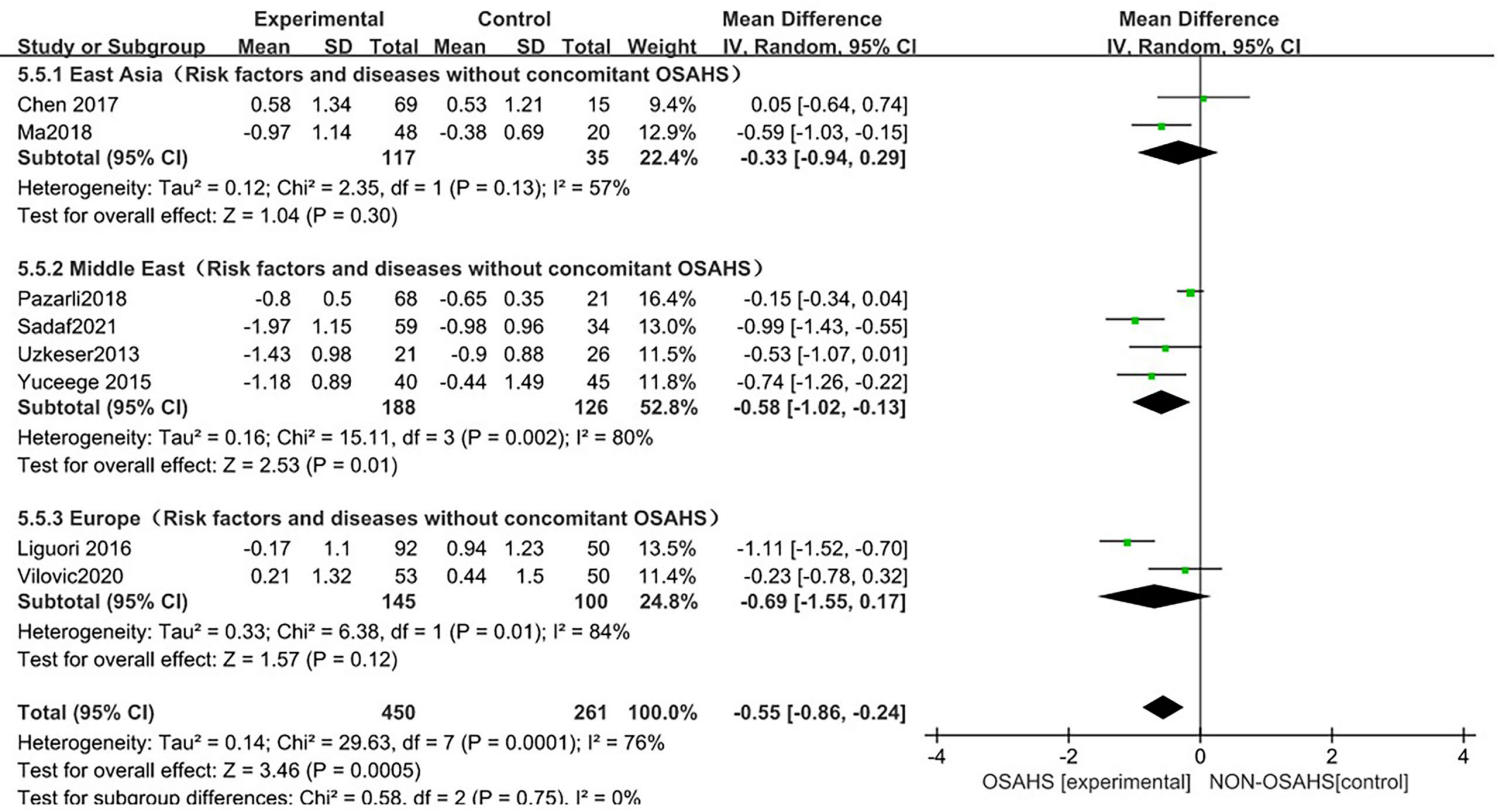
Forest plot of lumbar spine T-score “regional grouping” subgroup analysis between OSAHS group and control group (after excluding osteoporosis-related risk factors).

In the regional subgroup analysis (after the exclusion of osteoporosis-related risk factors), (**Figure 10**), 8 studies were included (3, 6, 15, 18, 20–23), and the combined group heterogeneity was (P = 0.0001, I^2^ = 76%), indicating a high degree of statistical heterogeneity. In the subgroup analysis, the grouping heterogeneity in East Asia was (P = 0.13, I^2^ = 57%), that in the Middle East was (P = 0.13, I^2^ = 57%). = 0.002, I^2^ = 80%), and that in Europe was (P = 0.01, I^2^ = 84%), so a random effect model was used; after the regional subgroup analysis, the heterogeneity of OSAHS and lumbar spine T-score correlation analysis was lower than before, among which the heterogeneity of East Asian grouping was reduced to moderate heterogeneity. The results of the subgroup analysis showed that, compared with the control group, the OSAHS group had a statistically significant difference in the Middle East group (MD = -0.58, 95% CI: -1.02~-0.13, Z = 2.53, P = 0.01); in the East Asian group (MD = -0.33, 95% CI: -0.94~0.29, Z = 1.04, P = 0.30), lumbar spine T-score was lower, but the difference was not statistically significant. In the Europe group (MD = -0.69, 95% CI: -1.55~0.17, Z = 1.57, P = 0.12), the lumbar spine T-score was lower, and the difference was also not statistically significant. The combined effect size of regional grouping (MD = -0.55, 95% CI: -0.86~-0.24, Z = 3.46, P < 0.001), the lumbar spine T-score of the OSAHS group was lower, After excluding the risk factors for osteoporosis, the results remained stable and the difference was statistically significant.

The forest plot analysis of OSAHS and lumbar spine T-score studies indicated that OSAHS is associated with lumbar spine T-score, OSAHS is a risk factor for lumbar spine T-score reduction, and the severity of OSAHS may be related to the lumbar spine T-score.

### 3.4 Sensitivity analysis

In this study, a sensitivity analysis was performed for the results with high heterogeneity. In the sensitivity analysis of the incidence of osteoporosis, lumbar bone mineral density, and lumbar spine T-score between OSAHS and the control group, the results and studies were combined after excluding any literature. There was no significant change in heterogeneity.

### 3.5 Publication bias

**Figure 11:**
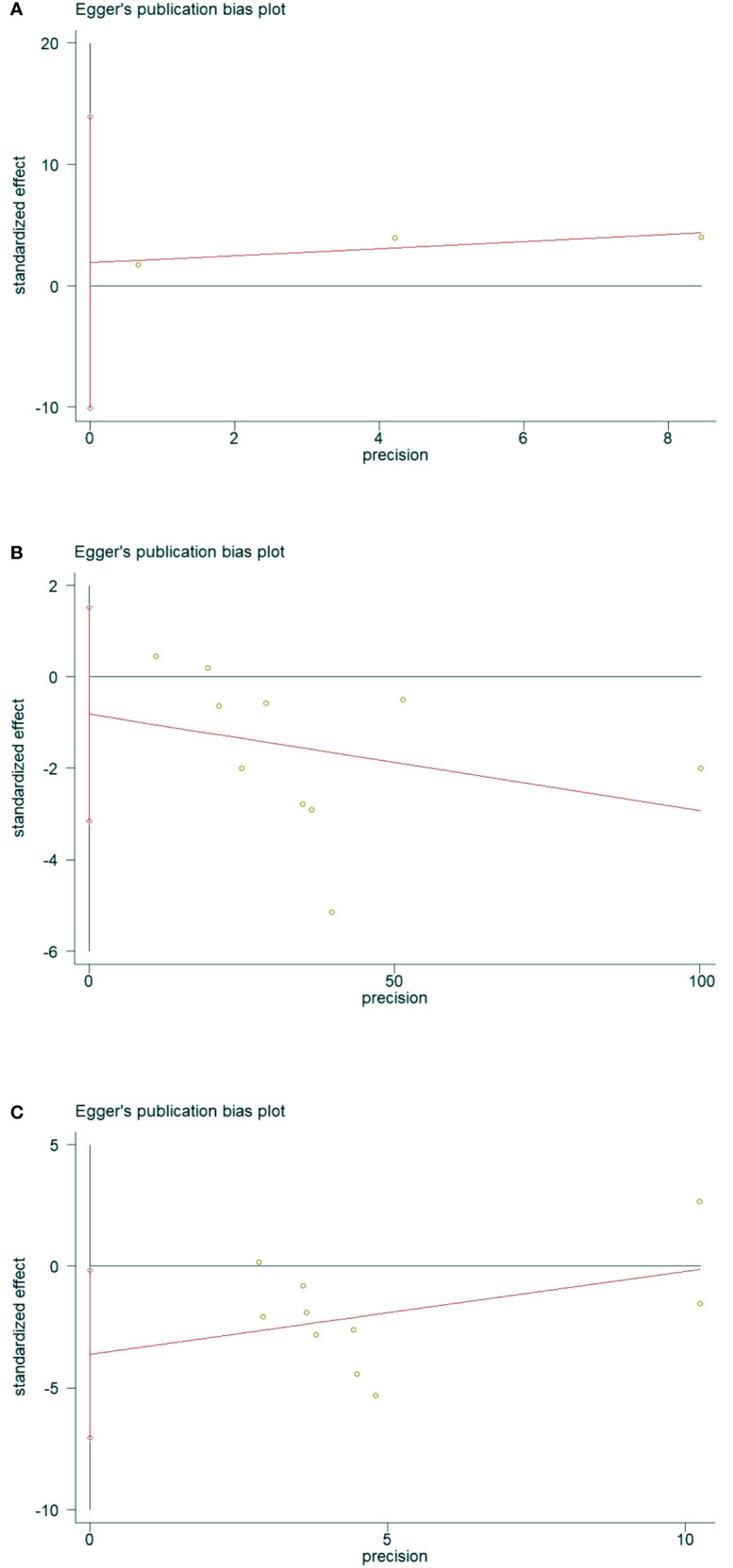
Data plot of Egger’s test for various studies included in the literature.(Note: **(A)** Data plot of Egger’s test for Osteoporosis incidence. **(B)** Data plot of Egger’s test for lumbar spine BMD. **(C)** Data plot of Egger’s test for lumbar spine T-score.).

The presence of publication bias was assessed using Egger’s method (**Figure 11**). There were 3 literatures related to OSAHS and the incidence of osteoporosis in the control group, P = 0.291>0.05 (**Figure 11A**), and the results indicated that there was no publication bias; there were 10 literatures related to OSAHS and the bone mineral density of the lumbar spine in the control group, P = 0.433 >0.05 (**Figure 11B**), the results suggest that there is no publication bias. There are 10 related literatures about lumbar spine T-score between OSAHS and control group, P=0.042<0. 05 (**Figure 11C**), the results suggest that there is mild publication bias, there may be reasons:1.The number of literatures included in our meta-analysis is small, which is easy to cause certain bias.2. Some study populations combined with other diseases may also have certain biases, which was further confirmed by our subgroup analysis.

## 4 Discussion

Osteoporosis is a common human skeletal disease characterized by osteopenia, microarchitectural deterioration, and fragility fractures (28). According to World Health Organization (WHO) standards, it is estimated that 15% of postmenopausal Caucasian women in the United States and 35% of women over the age of 65 have significant osteoporosis. One in two Caucasian women will experience an osteoporotic fracture at some point in their life. As early as 1994, a study showed that fragility fracture patients received more than 400,000 hospitalizations and more than 2.5 million doctor visits each year, causing a serious economic burden (29). Tomiyama et al. (30) first reported the correlation between OSAHS and abnormal bone metabolism in 2008. They studied the abnormal bone metabolism of 50 OSAHS patients and found that compared with the control group, a marker of bone resorption (urinary Type I collagen cross-linked C-terminal peptide) was significantly increased in the OSAHS group, and the elevated bone resorption marker levels decreased somewhat after three months of continuous positive airway pressure therapy. Subsequently, experimental and epidemiological studies have continuously explored the relationship between OSAHS and osteoporosis, BMD, and T-score, and its possible mechanism, but the results have not agreed.

Sikarin,Wang et al. (31, 32) conducted a meta-analysis of the correlation between OSAHS and bone marrow.Too few studies were included in the Sikarin’s analysis, and Sensitivity analysis, meta-regression, and publication bias were not performed.Wang’s analysis is mainly based on the Chinese population, so the research objects may not be representative of the general population, and there may be selection bias. The two meta-analyses did not conduct a global multi-regional population study, nor did further analysis based on the severity of OASHS, and the analysis indicators were relatively single. Therefore, in response to these problems, we conducted an update of the meta-analysis of the correlation between OSAHS and bone marrow.

This meta-analysis included 15 studies. The included studies were all high-quality studies with 6 stars and above according to the NOS quality evaluation.The results of the meta-analysis on the correlation between OSAHS and osteoporosis showed that in males, females, middle-aged people (40~65 years old) and elderly people (> 65 years old), patients with OSAHS had osteoporosis. Although the incidence of osteoporosis is high, only three articles were included in the literature, so more research is needed to further stabilize the results; in addition, the study population included in the literature may have been combined with old age, hypertension, diabetes, cardiovascular disease, COPD, etc., which may have affected the osteoporosis risk factors, and there is currently no prevalence data to exclude the relevant risk factors; thus, further investigation and analysis cannot be carried out, and the results are not stable.

BMD is an important indicator reflecting bone mineral content per unit area. It is mainly used to detect the osteoporosis degree, predict the risk of fracture, and provide a strong laboratory test basis for fractures caused by osteoporosis. BMD is clinically the gold standard for the diagnosis of osteoporosis (33). In recent years, many studies have focused on the relationship between OSAHS and BMD. Tomiyama, Sforza, and Chen et al. (7, 18, 30) found that compared with the control group, OSAHS patients had significantly higher BMD levels; and more studies, including Liguori, Uzkeser, Yuceege, Terzi, Qiao, Pazarli, Ma, and Vilovic et al. (3, 6, 15, 16, 19–22) found that compared with the control group, the bone marrow of OSAHS patients was significantly higher than that of the control group. In order to further confirm the relationship between OSAHS patients and BMD, we conducted a meta-analysis of the correlation between OSAHS and lumbar spine BMD which showed that compared with the control group, the OSAHS group had lower lumbar spine BMD. After further subgroup analysis, the combined effect size still confirms that the OSAHS group has lower lumbar spine BMD compared with the control group. In the subgroup analysis of AHI grouping with OSAHS diagnostic criteria, compared with the control group, the lumbar spine BMD of the OSAHS AHI > 5~10 events/h group and AHI > 15 events/h group were decreased, and the difference was statistically significant. The effect size of lumbar spine BMD in the OSAHS diagnostic criteria AHI > 15 events/h group was higher than that of the OSAHS diagnostic criteria AHI > 5~10 events/h group. Compared with the AHI > 5~10 events/h group, the risk of lumbar spine BMD decline is higher, so the severity of lumbar spine BMD may be related. In the regional subgroup analysis (after excluding risk factors related to osteoporosis), the results showed that compared with the control group, the OSAHS group had lower lumbar spine BMD in the East Asian group, Middle East group, and Europe group, but the differences were not statistically significant. After subgroup analysis, the heterogeneity of the studies could be further reduced, and the research bias was also reduced, indicating that the conclusions of this study are more reliable.

Regarding the possible mechanism of the association between OSAHS and decreased BMD: 1. OSAHS may lead to a state of vitamin D deficiency and induce secondary hyperparathyroidism, which may lead to bone demineralization and decreased BMD (34); 2. Hypoxia is closely related to changes in bone turnover, and recent *in vitro* studies have shown that lower nighttime oxygen levels are a feature of OSAHS, while hypoxia promotes osteoclast formation and activity while inhibiting osteoblast function, thus determining bone resorption (35, 36).

The T-score is also an important basis for detecting the degree of osteoporosis. According to the results of BMD and the WHO standard, patients are divided into three groups: normal BMD (T-score > -1.0 SD), osteopenia (T-score -1.0 to -2.5 SD) and osteoporosis (T-score < -2.5 SD) (37). In recent years, many studies have focused on the relationship between OSAHS and lumbar spine T-score. Sforza, Chen et al. (7, 18) showed that compared with the control group, the lumbar spine T-score level of OSAHS patients was significantly higher; and more studies by Liguori, Uzkeser, Yuceege, Wang, Terzi, Qiao, Pazarli, Ma and Vilovic et al. (3, 6, 15–17, 20–23) showed that compared with the control group, the lumbar spine T-score level of OSAHS patients was significantly lower. In order to further confirm the relationship between OSAHS patients and lumbar spine T-score levels, we conducted a meta-analysis of the correlation between OSAHS and lumbar spine T-score levels which showed that compared with the control group, the OSAHS group had lower lumbar spine T-score levels. After excluding the related factors of osteoporosis, further subgroup analysis was performed, and the combined effect size still confirmed that the lumbar spine T-score level was lower in the OSAHS group compared with the control group.

In the subgroup analysis of AHI grouping with OSAHS diagnostic criteria (after excluding osteoporosis-related risk factors), compared with the control group, OSAHS diagnostic criteria AHI > 5~10 events/h group and AHI > 15 events/h group, the lumbar spine T-score of all groups decreased, and the difference was statistically significant. The effect size of the lumbar spine T-score in the OSAHS diagnostic criteria AHI > 15 events/h group was higher than that in the OSAHS diagnostic criteria AHI > 5~10 events/h group, and the risk of lumbar spine T-score decline was higher than that in the AHI > 5~10 events/h group, indicating that the severity of OSAHS may be related to the lumbar spine T-score. In the regional subgroup analysis (after excluding risk factors related to osteoporosis), the results showed that compared with the control group, the OSAHS group had lower lumbar spine BMD in the East Asian group, Middle East group, and Europe group, but only the Middle East subgroup was statistically significant. In conclusion, compared with the control group, the OSAHS group had lower lumbar spine T-score levels in the OSAHS diagnostic criteria AHI > 5~10 events/h group, AHI > 15 events/h group and Middle East group. The heterogeneity and research bias can be further reduced, indicating that the conclusions of this study are more reliable. At present, there is a lack of research that clearly clarifies the relationship between T-score and BMD and osteoporosis. However, because T-score is scored according to BMD, the possible mechanism of the correlation between OSAHS and T-score reduction can also be understood.

This study has certain limitations: first, the number of included studies on the relationship between OSAHS and osteoporosis was small, and combined with the related risk factors of osteoporosis, the results were not stable, so more research is needed to further stabilize the study. Second, the diagnostic methods and grading methods of OSAHS in each study were slightly different, and the study populations were from different ethnic groups, which may have led to greater heterogeneity in the results. Third, osteoporosis is more common in women (38), but there were fewer women OSAHS patients in our meta-analysis, which may have generated a selection bias. Fourth, the study sample size was relatively small compared to a large, multicentric, randomized controlled trial. Fifth, the quality of some included literature was not very high, and there may have been selection bias. Therefore, the conclusions should be interpreted with caution.

In conclusion, the results of this study suggest that OSAHS patients have a higher incidence of osteoporosis, and both lumbar spine BMD and lumbar spine T-score are reduced. The severity of AHI may be related to lumbar spine BMD and lumbar spine T-score. Understanding the incidence of osteoporosis in patients with OSAHS and the effect of OSAHS on lumbar spine BMD and T-score provides medical evidence. However, a homogeneous and large-scale prospective study with further adjustment for factors such as age and related diseases affecting osteoporosis is still needed to clarify whether OSAHS is a risk factor for osteoporosis and whether OSAHS has an effect on lumbar spine BMD and T-score. Many drugs have been developed to treat osteoporosis (39), and patients should receive treatment if they have osteoporosis, and should be treated with preventive measures if they have osteopenia. Obviously, prevention is much better than treatment. Through the meta-analysis of this paper, it can be concluded that the effective management of OSAHS can effectively reduce the risk of osteoporosis.

## Data availability statement

The raw data supporting the conclusions of this article will be made available by the authors, without undue reservation.

## Author contributions

JHL, CW, ZPZ, ZZZ, XC and YZ are the guarantor of the manuscript and take responsibility for the content of this manuscript. JHL, QY, RC and CW contributed to the design of the study. HC, JZ, JYL and HZL were involved in the data analysis. ZPZ, JYL, HWL and RC contributed to the acquisition of primary data. ZPZ, CW and RC wrote the initial draft of the manuscript. QY, RC and JHL contributed significantly to the revision of the manuscript. All authors read and approved the final manuscript.

## Funding

This study was funded by the Natural Science Foundation of Guangdong Province (2021A1515011373).

## Acknowledgments

We would also like to thank Professor Nanshan Zhong from State Key Laboratory of Respiratory Disease for the constructive advice he gave.

## Conflict of interest

The authors declare that the research was conducted in the absence of any commercial or financial relationships that could be construed as a potential conflict of interest.

## Publisher’s note

All claims expressed in this article are solely those of the authors and do not necessarily represent those of their affiliated organizations, or those of the publisher, the editors and the reviewers. Any product that may be evaluated in this article, or claim that may be made by its manufacturer, is not guaranteed or endorsed by the publisher.
